# Improved Theory of the Effective Dipole Moments and Absolute Line Strengths of the XY_2_ Asymmetric Top Molecules in the *X*^2^*B*_1_ Doublet Electronic States

**DOI:** 10.3390/ijms241612734

**Published:** 2023-08-12

**Authors:** Oleg Ulenikov, Elena Bekhtereva, Olga Gromova, Aleksei Kakaulin, Christian Sydow, Sigurd Bauerecker

**Affiliations:** 1 Research School of High-Energy Physics, National Research Tomsk Polytechnic University, 634050 Tomsk, Russia; bextereva@tpu.ru (E.B.); olgerda@tpu.ru (O.G.); ank41@tpu.ru (A.K.); 2 Institut für Physikalische und Theoretische Chemie, Technische Universität Braunschweig, D-38106 Braunschweig, Germany; christian.sydow@tu-braunschweig.de

**Keywords:** asymmetric top molecules in non-singlet electronic states, spin–rotation interactions, absolute line strengths, effective dipole moment operator

## Abstract

A new effective dipole moment model for the XY_2_ ($C_{2v}$−symmetry) molecule in a doublet electronic state is derived that includes (as special cases) all currently known models of effective dipole moments for such types of molecules, and allows us to take into account the influence of spin–rotation interactions on the effective dipole moment operator that were not considered in the preceding studies. Necessary for the analysis of absolute line strengths, the matrix elements of this dipole moment operator are derived. A comparison with the previous analog models is made and discussed. The efficiency of the obtained results is illustrated, which have been applied to a set of the “forbidden” $\Delta K_{a}=2$ transitions of the $\nu_{3}$ band of the OClO free radical molecule.

## 1. Introduction

The problem of the best possible description of absolute strengths of molecular quantum transitions is one of the most important problems of physical chemistry because of numerous applications in chemical physics itself (e.g., in the determination of an intramolecular multi-dimensional dipole moment supersurface, unimolecular reaction rate theory, fundamental biomolecular reaction dynamics, etc.) as well as in a number of applied problems of the earth–atmosphere, planetology, astrophysics and astrochemistry, industry, etc. This problem was discussed in the literature many times for different types of molecules (see, e.g., Refs. [1,2,3,4,5]). In the present case, one of the most difficult problems to solve is associated with the so-called asymmetric top molecules; Refs. [6,7,8]. As an illustration, we mention here the basic study by Flaud and Camy-Peyret [1] where correct effective dipole moment operators for different types of ro-vibrational bands of the XY_2_ ($C_{2v}$-symmetry) molecule and their corresponding matrix elements on the ro-vibrational wave functions have been derived. The general results of that study were successfully used by many authors for the analysis of different XY_2_ ($C_{2v}$) molecules, and also for more complicated asymmetric top molecules (not having the possibility to refer here to all these studies, we mention only a few of them—refs. [9,10,11,12,13]—which have been fulfilled by the authors of this paper during recent years).

It should be noted that the basic paper [1] and the further above mentioned studies are dealing with asymmetric top molecules in singlet electronic states. However, even the simplest from the asymmetric top molecules (namely, the XY_2_ one with ($C_{2v}$-symmetry) ones) can be presented in nature not only in a singlet state but also in multiplet electronic states as well (the NO_2_ and ClO_2_ free radical molecules in the $X^{2}B_{1}$ electronic ground state can be mentioned, for example). The theory and the matrix elements being necessary for calculations of effective dipole moment operators for such molecules differ considerably in some aspects from the basic results of Ref. [1]. The corresponding theory and results for such molecules have been presented in the literature beginning from the eighties of the twentieth century (see, Refs. [14,15,16,17]). However, up to now, not all crucial effects and interactions are completely and correctly taken into account and described. Namely, it is evident that not only pure rotational centrifugal effects but also centrifugal effects that are caused by the spin–rotation interactions should be taken into account. In particular, it is clear (see, e.g., [18,19,20,21,22,23,24,25,26,27,28,29,30,31,32,33] and our recent studies [34,35,36] where the higher order spin–rotational effects were taken into account) that influences of both the pure rotational and spin–rotational centrifugal effects on the spin–ro-vibrational structure of the discussed type of molecules are comparable in size. Furthemore, related to the absolute strengths of spin–ro-vibrational transitions, up to now, the influence of spin–rotational interactions on the absolute transition strengths have been taken into account only via wave functions, which are eigen functions of the effective Hamiltonian of the considered vibrational band. The dependence of the effective dipole moment operator on the spin–rotational centrifugal effects has never been considered despite the obvious fact that neglect of similar effects in the effective Hamiltonian leads to an increase in the error by several tens of times. In the present study, we intend to fill this gap and derive an effective dipole moment operator of the XY_2_ ($C_{2v}$) molecule in a doublet electronic state, taking into account its dependence on the spin–rotational centrifugal effects also. To make the discussion more clear for the reader, we consider the problem of obtaining the effective dipole moment operator and the determination of its matrix elements for the pure rotational problem in Section 2 as a starting point. The main ideas and steps of discussion in Section 2 are then used in Section 3 and Section 4 for the analogous analysis of an effective dipole moment operator and absolute line intensities for the ro-vibrational problem (without spin–rotational interactions; Section 3). This is followed by the discussion of a model that takes into account the presence of spin–rotational interactions in the wave functions, but omits both rotational and spin–rotational effects in the effective dipole moment (Section 4). Finally, Section 5 presents results that are produced by the complete consideration of both pure rotational and spin–rotational centrifugal distortion effects in the wave functions and in the effective dipole moment operator.

## 2. Absolute Intensity of an Isolated Line of the XY_2_ ($C_{2v}$) Molecule in a Singlet Electronic State: Rotational Transitions

It is well known that (in the absence of an external field) the strength of transition from the quantum state $|\psi_{i}\rangle$ to the quantum states $|\psi_{f}\rangle$ is obtained as [1,14]:

(1) $$\begin{array}{c} S_{\nu_{0}}^{N}=\frac{8\pi^{3}\nu_{0}}{4\pi \epsilon_{0}3hc}\left[1-\exp\left(-\frac{hc\nu_{0}}{k_{B}T}\right)\right]\frac{g_{i}}{Z(T)}\exp\left(-\frac{E_{i}}{k_{B}T}\right)R_{i}^{f}, \end{array}$$

where [37,38]

(2) $$\begin{array}{c} R_{i}^{f}=\sum_{A}|\langle \psi_{i}|P_{A}|\psi_{f}{\rangle |}^{2}. \end{array}$$

In Equations (1) and (2), $|\psi_{i}\rangle$ and $|\psi_{f}\rangle$ are wavefunctions of the lower and upper states of a molecule; $\tilde{\nu_{0}}=\left(E_{f}-E_{i}\right)/hc$; $E_{i}$ and $E_{f}$ are the upper and the lower ro-vibrational energies of the transition; $g_{i}$ and Z(T) are the degeneracy due to the nuclear spin of the lower $|\psi_{i}\rangle$ state and the partition function, which depends on the temperature *T*; the operators $P_{A}$ (A=X,Y or *Z*) are the three components of the dipole moment of a molecule in the space-fixed coordinate system (SFS)—see, e.g., [37,38]—and, being dependent in the general case on the instantaneous distances between the nuclei, they can be written as:

(3) $$\begin{array}{c} P_{A}=\sum_{\alpha }k_{A\alpha }\left(\mu_{\alpha }^{e}+\sum_{\lambda }\mu_{\alpha }^{\lambda }q_{\lambda }+\sum_{\lambda ,\nu \geq \lambda }\mu_{\alpha }^{\lambda \nu }q_{\lambda }q_{\nu }+\ldots \right). \end{array}$$

Here, $k_{A\alpha }$ are elements of the direction cosines matrix [39]; $\mu_{\alpha }^{e}$ are the components of the permanent (equilibrium) dipole moment of a molecule in the molecule-fixed coordinate system (MFS); $q_{\lambda }$/$q_{\nu }$ are vibrational normal dimensionless coordinates [7,7] of a molecule; and $\mu_{\alpha }^{\lambda }$, $\mu_{\alpha }^{\lambda \nu }$, … are the parameters that describe the dependence of the dipole moment components $\mu_{\alpha }$ in the MFS on the normal vibrational coordinates (in the first step of discussion in this section, we will take into account the first term only in Equation (3) and consider rotational transitions only).

All the values in Equation (1) are usually known, and the only problem is the determination of the matrix elements, Equation (2). In this case, it is necessary to note that the $|\psi_{i}\rangle$ and/or $|\psi_{f}\rangle$ states can be nondegenerate and/or degenerate. In the second case, the $R_{i}^{f}$ value should be changed by

(4) $$\begin{array}{c} R_{i}^{f}=\sum_{\alpha \beta }\sum_{A}|\langle \psi_{i}^{\alpha }|P_{A}|\psi_{f}^{\beta }{\rangle |}^{2}, \end{array}$$

where indexes α and β numerate sets of degenerate states. Because we speak here about transitions between rotational states only, it is suitable to use the functions $|\psi_{i}\rangle$ and $|\psi_{f}\rangle$ in the form of superpositions of the known |Jkm〉 functions (see [39,40,41,42]), which are transformed in accordance with the $D^{\left(J\right)}$ irreducible representation of the SO(3) symmetry group, and to use the equations $R_{\left(Jk\right)}^{\left(\tilde{J}\;\tilde{k}\right)}$:

(5) $$\begin{array}{c} R_{\left(Jk\right)}^{\left(\tilde{J}\;\tilde{k}\right)}=\sum_{m\tilde{m}}\sum_{A}\left|\langle Jkm\right|P_{A}|\tilde{J}\;\tilde{k}\;\tilde{m}{\rangle |}^{2} \end{array}$$

instead of $R_{i}^{f}$; Equation (4).

Let us consider now the matrix element $\langle Jkm|P_{Z}|\tilde{J}\;\tilde{k}\;\tilde{m}\rangle$ (A=Z) in Equation (5) and take into account that the three components $P_{X}$, $P_{Y}$ and $P_{Z}$, of the dipole moment operator **P** (remember that only the first terms in Equation (3) are taken into account in this section) can be expressed (see, e.g., [40]) in the form of three components of the irreducible first rank tensor $P_{S}^{\left(1\right)}$ S=0,±1:

(6) $$\begin{array}{c} P_{0}^{\left(1\right)}=P_{Z},\;\;\;\;\mathrm{and}\;\;\;\;P_{\pm 1}^{\left(1\right)}=\mp \frac{1}{\sqrt{2}}\left(P_{X}\mp iP_{Y}\right). \end{array}$$

In accordance with the known formulas of the irreducible tensorial sets theory [39,41,42], one can write:

(7) $$\begin{array}{ccc} \langle Jkm|P_{Z}|\tilde{J}\;\tilde{k}\;\tilde{m}\rangle =\langle Jkm|P_{0}^{\left(1\right)}|\tilde{J}\;\tilde{k}\;\tilde{m}\rangle & = & {\left(2J+1\right)}^{-1/2}C_{\tilde{J}\tilde{m},10}^{Jm}<Jk\left\|P^{\left(1\right)}\right\|\tilde{J}\;\tilde{k}>\\ & \equiv & {\left(2J+1\right)}^{-1/2}C_{\tilde{J}\tilde{m},10}^{Jm}<Jk\left|P_{Z}\right|\tilde{J}\;\tilde{k}> \end{array}$$

and

(8) $$\begin{array}{ccc} \langle Jkm|P_{X}|\tilde{J}\;\tilde{k}\;\tilde{m}\rangle & = & \frac{1}{\sqrt{2}}\langle Jkm|\left(P_{-1}^{\left(1\right)}-P_{1}^{\left(1\right)}\right)|\tilde{J}\;\tilde{k}\;\tilde{m}\rangle \\ & = & \frac{1}{\sqrt{2(2J+1)}}\left\{C_{\tilde{J}\tilde{m},1-1}^{Jm}-\;C_{\tilde{J}\tilde{m},11}^{Jm}\right\}<Jk\left|P_{Z}\right|\tilde{J}\;\tilde{k}>, \end{array}$$

(9) $$\begin{array}{ccc} \langle Jkm|P_{Y}|\tilde{J}\;\tilde{k}\;\tilde{m}\rangle & = & -\frac{i}{\sqrt{2}}\langle Jkm|\left(P_{-1}^{\left(1\right)}+P_{1}^{\left(1\right)}\right)|\tilde{J}\;\tilde{k}\;\tilde{m}\rangle \\ & = & -\frac{i}{\sqrt{2(2J+1)}}\left\{C_{\tilde{J}\tilde{m},1-1}^{Jm}+\;C_{\tilde{J}\tilde{m},11}^{Jm}\right\}<Jk\left|P_{Z}\right|\tilde{J}\;\tilde{k}>. \end{array}$$

If one now uses Equations (7)–(9) in Equation (5) and takes into account [39]:

(10) $$\begin{array}{c} \sum_{m\tilde{m}}\sum_{S}C_{\tilde{J}\tilde{m},1S}^{Jm}C_{\tilde{J}\tilde{m},1S}^{Jm}=\left(2J+1\right), \end{array}$$

then it is not difficult to show that

(11) $$\begin{array}{c} R_{\left(Jk\right)}^{\left(\tilde{J}\;\tilde{k}\right)}=\left|\langle Jk\right|P_{Z}|\tilde{J}\;\tilde{k}{\rangle |}^{2}, \end{array}$$

or

(12) $$\begin{array}{c} R_{\left(Jk\right)}^{\left(\tilde{J}\;\tilde{k}\right)}=\left|\langle Jk\right|\sum_{\alpha }k_{Z\alpha }|\tilde{J}\;\tilde{k}{\rangle |}^{2}, \end{array}$$

and the nonzero matrix elements are [43]:

(13) $$\begin{array}{c} <Jk|k_{Zz}|Jk>=k{\left\{\frac{\left(2J+1\right)}{J(J+1)}\right\}}^{1/2}, \end{array}$$

(14) $$\begin{array}{ccc} & & <Jk|k_{Zx}|Jk\pm 1>=\pm <Jk|ik_{Zy}|Jk\pm 1>\\ & & =\frac{1}{2}{\left\{\frac{\left(2J+1)(J\mp k)(J\pm k+1\right)}{J(J+1)}\right\}}^{1/2}; \end{array}$$

(15) $$\begin{array}{c} <Jk|k_{Zz}|J+1k>={\left\{\frac{\left(J+k+1)(J-k+1\right)}{\left(J+1\right)}\right\}}^{1/2}, \end{array}$$

(16) $$\begin{array}{ccc} & & <Jk|k_{Zx}|J+1k\pm 1>=\pm <Jk|ik_{Zy}|J+1k\pm 1>\\ & & =\mp \frac{1}{2}{\left\{\frac{\left(J\pm k+1)(J\pm k+2\right)}{\left(J+1\right)}\right\}}^{1/2}; \end{array}$$

(17) $$\begin{array}{c} <Jk|k_{Zz}|J-1k>={\left\{\frac{\left(J+k)(J-k\right)}{J}\right\}}^{1/2}, \end{array}$$

(18) $$\begin{array}{ccc} & & <Jk|k_{Zx}|J-1k\pm 1>=\pm <Jk|ik_{Zy}|J-1k\pm 1>\\ & & =\pm \frac{1}{2}{\left\{\frac{\left(J\mp k)(J\mp k-1\right)}{J}\right\}}^{1/2}. \end{array}$$

## 3. Absolute Intensity of an Isolated Line of the XY_2_ ($C_{2v}$) Molecule in a Singlet Electronic State: Ro-Vibrational Transitions

Let us consider now the physically more correct model with the dipole moment operators $P_{A}$, which has the whole form of Equation (3), and remember some information from the general effective Hamiltonian theory [7,44,44]. Let us also assume that we want to solve the Schrödinger equation

(19) $$\begin{array}{c} H|\psi_{i}\rangle =E_{i}|\psi_{i}\rangle \end{array}$$

with a rotation–vibration Hamiltonian *H* (see, e.g., [7,8]), and are not interested in a full set of eigenvalues of the Hamiltonian *H*, but only in a subset of ro-vibrational states |v,R(Jk)〉, which are connected with a separate vibrational state (v). In this case, in accordance with the “effective Hamiltonian theory”, it is suitable to change Equation (19) (which, in the general case, is a very complicated problem, if at all possible in principle) by the Schrödinger equation

(20) $$\begin{array}{c} \tilde{H}|v^{\prime },R_{i}\left(Jk\right)\rangle =\delta_{vv^{\prime }}E_{R_{i}\left(JK\right)}^{\left(v\right)}|v,R_{i}\left(Jk\right)\rangle =\delta_{vv^{\prime }}E_{Ri(Jk)}^{\left(v\right)}|v\rangle |R_{i}\left(Jk\right)\rangle , \end{array}$$

whose set of Eigen ro-vibrational energies $E_{R_{i}\left(Jk\right)}^{\left(v\right)}$ totally coincides with a corresponding subset of eigenvalues of Equation (19); the ∣v〉 and $|R_{i}\left(Jk\right)\rangle$ in Equation (20) are pure vibrational and rotational functions (the latter depend on quantum numbers *J* and *k* of the MFS). Such an operator $\tilde{H}$, which is usually presented in the form of

(21) $$\begin{array}{c} \tilde{H}=G^{+}HG \end{array}$$

(*G* is a unitary ro-vibration operator) is called an “effective rotational operator” of the (v) vibrational state of a molecule (see details, e.g., in [7,8,44]). Here, evidently, the $|v,R_{i}\left(Jk\right)\rangle$ functions from Equation (20) are connected with the corresponding $|\psi_{i}\rangle$ functions from Equation (19) as

(22) $$\begin{array}{c} |\psi_{i}\rangle =G|v,R_{i}\left(Jk\right)\rangle . \end{array}$$

Let us take into account that the rotational parts $|R_{i}\left(Jk\right)\rangle$ of the eigen functions $|v\rangle |R_{i}\left(Jk\right)\rangle$ of the effective operator $\tilde{H}$ can be, evidently, presented in the form of superpositions of the functions $|Jk^{\prime }\rangle$, which are presented in Equation (11):

(23) $$\begin{array}{c} |R_{i}\left(Jk\right)\rangle =\sum_{\tilde{k}}B_{J\tilde{k}}^{i}|J\tilde{k}\rangle . \end{array}$$

To construct new functions, now

(24) $$\begin{array}{c} |R_{i}\left(Jkm\right)\rangle =\sum_{k^{\prime }}B_{Jk^{\prime }}^{i}|Jk^{\prime }m\rangle , \end{array}$$

use the latter in Equation (2), and make the transformation analogous to the transformations from Section 2; then, it is not difficult to obtain the following form analogous to Equation (11), but, already for a ro-vibrational transition,

(25) $$\begin{array}{c} R_{\left(v,\;R_{i}\left(Jk\right)\right)}^{\left(\tilde{v},\;{\tilde{R}}_{j}\left(\tilde{J}\;\tilde{k}\right)\right)}=|\langle v,R_{i}\left(Jk\right)|\left\{G^{+}P_{Z}G\right\}|\tilde{v},{\tilde{R}}_{j}\left(\tilde{J}\;\tilde{k}\right)\rangle {|}^{2}. \end{array}$$

In turn, taking into account that $\sum_{v}|v\rangle \langle v|=1$, the operator $G^{+}P_{Z}G$ in Equation (25) can be re-written as

(26) $$\begin{array}{c} G^{+}P_{Z}G=\sum_{v^{\prime }\;{\tilde{v}}^{\prime }}|v^{\prime }\rangle \left(\langle v^{\prime }|G^{+}P_{Z}G|{\tilde{v}}^{\prime }\rangle \right)\langle \tilde{v^{\prime }}|. \end{array}$$

The pure rotational operator ${}^{\left(v^{\prime }-{\tilde{v}}^{\prime }\right)}P_{Z}=\langle v^{\prime }|G^{+}P_{Z}G|{\tilde{v}}^{\prime }\rangle$ is usually called an “effective dipole moment” operator for the $\left(\tilde{\nu }-\nu \right)$ band. Because, for the XY_2_ ($C_{2v}$) molecule, the symmetry of vibrational functions ∣v〉 can be $A_{1}$ or $B_{1}$, only two types of the “effective dipole moment” operators can be realized, namely ${}^{\left(v^{\prime }-{\tilde{v}}^{\prime }\right)}P_{Z}^{A{}_{2}}$ for the parallel bands and ${}^{\left(v^{\prime }-{\tilde{v}}^{\prime }\right)}P_{Z}^{B{}_{2}}$ for the perpendicular bands. In both cases, from the general point of view (see, e.g., [8]), the ”effective dipole moment“ operator can be written as:

(27) $$\begin{array}{c} {}^{\left(v-\tilde{v}\right)}P_{Z}^{\gamma }=\frac{1}{2}{\left\{\sum_{\alpha }k_{Z\alpha }^{\Gamma },\sum_{p+q+r=0}^{\infty }\mu_{pqr}^{\left(v-\tilde{v}\right)}{\left(J_{x}^{p}J_{y}^{q}J_{z}^{r}+J_{z}^{r}J_{y}^{q}J_{x}^{p}\right)}^{\tilde{\Gamma }}\right\}}_{+}^{\gamma }, \end{array}$$

where we use the operator ${}^{\left(v-\tilde{v}\right)}P_{Z}^{\gamma }$ in the form of an anticommutator because of the requirement of its hermiticity; and the values $\mu_{pqr}^{\left(v-\tilde{v}\right)}$ are of different orders of the values of effective dipole moment parameters. First- and second-order $\mu_{pqr}^{\left(v-\tilde{v}\right)}$ parameters both for the parallel and perpendicular bands have been presented in [1], and, for the convenience of the reader, we simply reproduce results from [1] in Table 1 and Table 2.

## 4. Absolute Intensity of an Isolated Line of the XY_2_ ($C_{2v}$) Molecule in Doublet Electronic State: Spin–Rotational Transitions in the Model That Neglects Spin–Rotational Interactions in the Effective Dipole Moment Operator

Let us consider now an object that is more important for the present study, namely the XY_2_ ($C_{2v}$ symmetry) molecule in a doublet electronic state. To our knowledge, for a description of absolute strengths of spin–ro-vibrational transitions in such molecules, up to now, the modern chemical physics uses the model that takes into account the presence of spin–rotation interactions in the wave functions of the lower and upper states of the transition considered, but neglects the presence of spin–rotational interactions in the effective dipole moment operator (see, e.g., [14,15,16,17]). It looks rather inconsistent if one takes into account the following arguments: (a) the influence of the rotational centrifugal distortion effects on an effective dipole moment operator is always taken into account for molecules both in singlet and doublet electronic states (see, e.g., above-mentioned Refs. [1,14,15,16,17]); (b) as was discussed above, influences of both the pure rotational and spin–rotational centrifugal distortion effects on the spin–ro-vibrational energies of asymmetric top molecules in doublet electronic states are practically of the same orders of value; (c) in this respect, one can expect that taking into account spin–rotation interactions in an effective dipole moment operator can improve the accuracy of the description of absolute transition strengths in a doublet electronic state molecule by the same order as the pure rotational centrifugal effects improve the description of absolute transition strengths in a singlet electronic state molecule.

In this Section 4 and the next Section 5, following the scheme of transformation in the preceding Section 2, we discuss both models (without and with taking into account spin–rotation interactions in the effective dipole moment operator).

Following the traditional approach [40], the transition from the description of the absolute line strength of a molecule in a singlet electronic state to the corresponding description of a molecule in a doublet electronic state needs some changes in the general formula; Equation (5). Namely, it is necessary to change the pure rotational functions |Jkm〉 by the spin–rotational functions ${\left(|Jk\rangle ⊗|S\rangle \right)}_{km}^{N}$. In this case, in accordance with [39]:

(28) $$\begin{array}{ccc} & & {\left(\langle J|⊗\langle S|\right)}_{km}^{N}{\left(P_{Z}\equiv P^{\left(1\right)}\right)}_{0}^{1}{\left(|\tilde{J}\rangle ⊗|S\rangle \right)}_{\;\tilde{k}\;\tilde{m}}^{\tilde{N}}\\ & & \equiv {\left(\langle J|⊗\langle S|\right)}_{km}^{N}{\left(P^{\left(1\right)}⊗1\right)}_{0}^{1}{\left(|\tilde{J}\rangle ⊗|S\rangle \right)}_{\;\tilde{k}\;\tilde{m}}^{\tilde{N}}\\ & & ={\left(2N+1\right)}^{-1/2}C_{\tilde{N}\tilde{m},10}^{Nm}\left\{\begin{array}{ccc} J & S & N\\ \tilde{J} & S & \tilde{N}\\ 1 & 0 & 1 \end{array}\right\}{\left(2N+1\right)}^{1/2}{\left(2\tilde{N}+1\right)}^{1/2}\\ & & \times \sqrt{3}<Jk\|P^{\left(1\right)}\|\tilde{J}\;\tilde{k}><S\left\|1\right\|S>, \end{array}$$

where $<Jk\|P^{\left(1\right)}\|\tilde{J}\;\tilde{k}>$ can be taken from Equation (7), <S‖1‖S>=1, and $\left\{\begin{array}{ccc} J & S & N\\ \tilde{J} & S & \tilde{N}\\ 1 & 0 & 1 \end{array}\right\}$ are 9J−symbols of the SO(3) symmetry group [39]. Analogously (taking into account Equation (6)), one can obtain the corresponding matrix elements of the $P_{X}$ and $P_{Y}$ components of the dipole moment operator:

(29) $$\begin{array}{ccc} & & {\left(\langle J|⊗\langle S|\right)}_{km}^{N}P_{X}{\left(|\tilde{J}\rangle ⊗|S\rangle \right)}_{\tilde{k}\;\tilde{m}}^{\tilde{N}}\\ & & =\frac{1}{\sqrt{2}}{\left(\langle J|⊗\langle S|\right)}_{km}^{N}\left\{{\left(P^{\left(1\right)}⊗1\right)}_{-1}^{1}-{\left(P^{\left(1\right)}⊗1\right)}_{1}^{1}\right\}{\left(|\tilde{J}\rangle ⊗|S\rangle \right)}_{\tilde{k}\;\tilde{m}}^{\tilde{N}}\\ & & =\frac{1}{\sqrt{2}}\left(C_{\tilde{N}\tilde{m},1-1}^{Nm}-C_{\tilde{N}\tilde{m},11}^{Nm}\right)\left\{\begin{array}{ccc} J & S & N\\ \tilde{J} & S & \tilde{N}\\ 1 & 0 & 1 \end{array}\right\}{\left(2\tilde{N}+1\right)}^{1/2}\sqrt{3}<Jk\left\|P^{1}\right\|\tilde{J}\;\tilde{k}> \end{array}$$

and

(30) $$\begin{array}{c} {\left(\langle J|⊗\langle S|\right)}_{km}^{N}P_{Y}{\left(|\tilde{J}\rangle ⊗|S\rangle \right)}_{\tilde{k}\;\tilde{m}}^{\tilde{N}}\\ =\frac{-i}{\sqrt{2}}{\left(\langle J|⊗\langle S|\right)}_{km}^{N}\left\{{\left(P^{\left(1\right)}⊗1\right)}_{-1}^{1}+{\left(P^{\left(1\right)}⊗1\right)}_{1}^{1}\right\}{\left(|\tilde{J}\rangle ⊗|S\rangle \right)}_{\tilde{k}\;\tilde{m}}^{\tilde{N}}\\ =\frac{-i}{\sqrt{2}}\left(C_{\tilde{N}\tilde{m},1-1}^{Nm}+C_{\tilde{N}\tilde{m},11}^{Nm}\right)\left\{\begin{array}{ccc} J & S & N\\ \tilde{J} & S & \tilde{N}\\ 1 & 0 & 1 \end{array}\right\}{\left(2\tilde{N}+1\right)}^{1/2}\sqrt{3}<Jk\left\|P^{1}\right\|\tilde{J}\;\tilde{k}>. \end{array}$$

If one now uses the relations (28)–(30) in Equation (5) (but uses the functions ${\left(|J\rangle ⊗|S\rangle \right)}_{km}^{N}$ instead of functions |Jkm〉) and takes into account Equation (10), then the following result can be derived (the numerical values of the 9J−symbols in Equation (30) can be easily obtained on the basis of formulas from Ref. [39]):

(31) $$\begin{array}{ccc} R_{\left(NJk\right)}^{\left(\tilde{N}\;\tilde{J}\;\tilde{k}\right)} & & =3\left(2N+1\right)\left(2\tilde{N}+1\right){\left\{\begin{array}{ccc} J & S=1/2 & N\\ \tilde{J} & S=1/2 & \tilde{N}\\ 1 & 0 & 1 \end{array}\right\}}^{2}\left|\langle Jk\right|P_{Z}|\tilde{J}\;\tilde{k}{\rangle |}^{2}\\ & & \equiv \tilde{g}\left(NJ,\tilde{N}\tilde{J}\right)\left|\langle Jk\right|\left(P_{Z}\equiv \sum_{\alpha }k_{Z\alpha }\right)|\tilde{J}\;\tilde{k}{\rangle |}^{2}. \end{array}$$

Corresponding nonzero $\tilde{g}\left(NJ,\tilde{N}\tilde{J}\right)$ coefficients are presented in Table 3. It is interesting that, for values of the $\tilde{g}\left(NJ,\tilde{N}\tilde{J}\right)$ coefficient, the following relations are valid:

(32) $$\begin{array}{c} \tilde{g}\left(NJ,\tilde{N}\tilde{J}\right)=\frac{1}{2}g\left(NJ,\tilde{N}\tilde{J}\right), \end{array}$$

where $\tilde{g}\left(NJ,\tilde{N}\tilde{J}\right)$ are the analogous coefficients from Ref. [14]. This circumstance practically does not affect the result of the description of absolute spin–ro-vibrational transitions but nevertheless leads to different (changed by the factor close to $\sqrt{2}$) values of effective dipole moment parameters that can be derived from the analysis of experimental data. It is also important that, for any of the three multiplets ($\tilde{N}=N,N\pm 1$) in Table 3, sums of corresponding $\tilde{g}$−coefficients are equal to 1. This circumstance gives the possibility to interpret the values $\tilde{g}\left(NJ,\tilde{N}\tilde{J}\right)$ from Table 3 as coefficients of the distribution of the absolute strengths of the transition $\left|NJk\rangle \to \right|\tilde{N}\;\tilde{J}\;\tilde{k}\rangle$ between its spin–rotational components.

## 5. Absolute Intensity of an Isolated Line of the XY_2_ ($C_{2v}$) Molecule in Doublet Electronic State: Spin–Rotational Transitions: $P_{Z}$-Operator Depends on Molecular Vibrations

Let us consider now the more correct model of the dipole moment operator in the form of Equation (3), which depends on molecular vibrations, and connect it to the transformations and discussion from Section 3. It is necessary to take into account that the analogous form of Equation (20) should be the following (because the operator, Equation (21), depends not only on the rotational variables but on the spin variables as well):

(33) $$\begin{array}{ccc} \tilde{H}|v^{\prime },R_{i}\left(Nk,SJ\right)\rangle & & =\delta_{vv^{\prime }}E_{v,R_{i}\left(Nk,SJ\right)}^{\left(v\right)}|v,R_{i}\left(Nk,SJ\right)\rangle \\ & & =\delta_{vv^{\prime }}E_{v,R_{i}\left(Nk,SJ\right)}^{\left(v\right)}|v\rangle |R_{i}\left(Nk,SJ\right)\rangle , \end{array}$$

where $|v\rangle |R_{i}\left(Nk,SJ\right)\rangle$ are eigenfunctions of the effective spin–rotation Hamiltonian of Equation (20) type (for details concerning an effective spin–rotation Hamiltonian, see, e.g., our recent papers [34,35,36]). In this case, the basic Equation (5) should be also changed by

(34) $$\begin{array}{c} R_{\left(v,R_{i}\left(Nk,SJ\right)\right)}^{\left(\tilde{v},\;{\tilde{R}}_{j}\left(\tilde{N}\;\tilde{k},\;S\;\tilde{J}\right)\right)}=\sum_{m\tilde{m}\lambda }|{\left(\langle J|⊗\langle S|\right)}_{km}^{N}\langle v|{\left(G^{+}P^{\left(1\right)}G\right)}_{\lambda }^{1}|\tilde{v}\rangle {\left(|\tilde{J}\rangle ⊗|S\rangle \right)}_{\tilde{k}\;\tilde{m}}^{\tilde{N}}{|}^{2}, \end{array}$$

or (analogously to Equations (7)–(10)):

(35) $$\begin{array}{c} R_{\left(v,R_{i}\left(Nk,SJ\right)\right)}^{\left(\tilde{v},\;{\tilde{R}}_{j}\left(\tilde{N}\;\tilde{k},\;S\;\tilde{J}\right)\right)}=|{\left(\langle J|⊗\langle S|\right)}_{k}^{N}∥\langle v|{\left(G^{+}P^{\left(1\right)}G\right)}^{1}|\tilde{v}\rangle ∥{\left(|\tilde{J}\rangle ⊗|S\rangle \right)}_{\tilde{k}}^{\tilde{N}}{|}^{2}\;\;\\ =\;\;|{\left(\langle J|⊗\langle S|\right)}_{k}^{N}\sum_{\alpha }{\left\{\frac{1}{2}k_{Z\alpha }^{\Gamma },\sum_{p+q+r=0}^{\infty }{}^{\left(\alpha \right)}\mu_{pqr}^{\left(v-\tilde{v}\right)}{\left(J_{x}^{p}J_{y}^{q}J_{z}^{r}+J_{z}^{r}J_{y}^{q}J_{x}^{p}\right)}^{\tilde{\Gamma }}\;\;+\sum_{p+q+r=1}^{\infty }\sum_{\beta }^{x,y,z}{}^{\left(\alpha \right)}\mu_{pqr\beta }^{\left(v-\tilde{v}\right)}{\left(J_{x}^{p}J_{y}^{q}J_{z}^{r}S_{\beta }+S_{\beta }J_{z}^{r}J_{y}^{q}J_{x}^{p}\right)}^{\tilde{\Gamma }}\right\}}_{+}^{\gamma }{\left(|\tilde{J}\rangle ⊗|S\rangle \right)}_{\tilde{k}}^{\tilde{N}}{|}^{2}\;\;\\ =\;\;|{\left(\langle J|⊗\langle S|\right)}_{k}^{N}\sum_{\alpha }{\left\{\frac{1}{2}k_{Z\alpha }^{\Gamma },{}^{\left(\alpha \right)}\mu_{\mathrm{rot}}^{\tilde{\Gamma }}+{}^{\left(\alpha \right)}\mu_{\mathrm{sp}-\mathrm{rot}}^{\tilde{\Gamma }}\right\}}_{+}^{\gamma }{\left(|\tilde{J}\rangle ⊗|S\rangle \right)}_{\tilde{k}}^{\tilde{N}}{|}^{2}. \end{array}$$

Here, $\gamma =(\Gamma \times \tilde{\Gamma })$, and, in the right hand side of Equation (35), we omitted a term, which depends on the $S_{\alpha }$ operators only, because it can give only an insufficient addition to the main effective dipole moment parameters $\mu_{000}^{\left(v-\tilde{v}\right)}$.

Before further discussion, we would like to make the following remark concerning the main term $\sum_{\alpha }{}^{\alpha }\mu_{000}^{\left(v-\tilde{v}\right)}k_{Z\alpha }^{\Gamma }$. If one will come back to the formulas Equations (28)–(30) and use the general relation

(36) $$\begin{array}{c} {\left(\langle J|⊗\langle S|\right)}_{km}^{N}\left(P_{\lambda }^{\left(1\right)}\right){\left(|\tilde{J}\rangle ⊗|S\rangle \right)}_{\tilde{k}\;\tilde{m}}^{\tilde{N}}={\left(2N+1\right)}^{-1/2}C_{\tilde{N}\tilde{m},1\lambda }^{Nm}{\left(\langle J|⊗\langle S|\right)}_{k}^{N}∥P^{\left(1\right)}∥{\left(|\tilde{J}\rangle ⊗|S\rangle \right)}_{\tilde{k}}^{\tilde{N}}, \end{array}$$

then it is not difficult to obtain

(37) $$\begin{array}{ccc} & & \sum_{m,\tilde{m},\lambda }|{\left(\langle J|⊗\langle S|\right)}_{km}^{N}\left(P_{\lambda }^{\left(1\right)}\right){\left(|\tilde{J}\rangle ⊗|S\rangle \right)}_{\tilde{k}\;\tilde{m}}^{\tilde{N}}{|}^{2}=\;\;|{\left(\langle J|⊗\langle S|\right)}_{k}^{N}∥P^{\left(1\right)}∥{\left(|\tilde{J}\rangle ⊗|S\rangle \right)}_{\tilde{k}}^{\tilde{N}}{|}^{2}\\ & & =\;\;|{\left(\langle J|⊗\langle S|\right)}_{k}^{N}\left(\sum_{\alpha }k_{Z\alpha }\right){\left(|\tilde{J}\rangle ⊗|S\rangle \right)}_{\tilde{k}}^{\tilde{N}}{|}^{2}. \end{array}$$

Now, from a comparison of Equations (31) and (37), one can obtain the following relation:

(38) $$\begin{array}{c} {\left(\langle J|⊗\langle S|\right)}_{k}^{N}\left(\sum_{\alpha }k_{Z\alpha }\right){\left(|\tilde{J}\rangle ⊗|S\rangle \right)}_{\tilde{k}}^{\tilde{N}}=\sqrt{\tilde{g}\left(NJ,\tilde{N}\tilde{J}\right)}\;\;\langle Jk|\left(\sum_{\alpha }k_{Z\alpha }\right)|\tilde{J}\;\tilde{k}\rangle , \end{array}$$

whose right-side part is nothing else than the main part of the effective operator in Equation (35).

Because the $\mu_{\mathrm{rot}}^{\tilde{\Gamma }}$ operator in Equation (35) depends on the rotational operators only, then both itself and its matrix elements are not different from the corresponding results discussed in Section 5. With regard to the spin–rotational effects that are described by the $\mu_{\mathrm{sp}-\mathrm{rot}}^{\tilde{\Gamma }}$ operator and that have never been discussed in the literature earlier, their main parts (which are proportional to the products $R_{\alpha }S_{\beta }$) are considered below on the basis of the symmetry properties of the discussed molecule (here, and further in Section 5, we use the notation $R_{0}^{1}$, $R_{\mp 1}^{1}$ for the rotational angular momentum operators, which are written in the notation of the irreducible rotational sets theory). It is not difficult to show that, for the XY_2_ ($C_{2v}$) molecule, the $R_{\alpha }$ (α=x,y,z) components in the MFS are:

(39) $$\begin{array}{c} R_{x}=\frac{1}{\sqrt{2}}\left(R_{-1}^{\left(1\right)}-R_{1}^{\left(1\right)}\right)\in A_{2},\;\;\;\;\;R_{y}=-\frac{i}{\sqrt{2}}\left(R_{-1}^{\left(1\right)}+R_{1}^{\left(1\right)}\right)\in B_{1},\;\;\;\mathrm{and}\;\;\;\;\;R_{z}=R_{0}^{\left(1\right)}\in B_{2}, \end{array}$$

where $A_{2}$, $B_{1}$, and $B_{2}$ are irreducible representations of the $C_{2v}$ point symmetry group.

First of all, taking into account the symmetry of the operators $R_{\alpha }$ and $S_{\beta }$ in the XY_2_ ($C_{2v}$ symmetry) molecule (evidently, the same as for the $R_{\alpha }$ operators, $S_{x}\in A_{2}$, $S_{y}\in B_{1}$, and $S_{z}\in B_{2}$), it is not difficult to obtain symmetrized combinations of different products of operators $J_{\alpha }$ and $S_{\beta }$. They are:

(40) $$\begin{array}{c} \left(R\cdot S\right)\equiv \sum_{\alpha }R_{\alpha }S_{\alpha }\in A_{1},\;\;\;\;\;R_{z}S_{z}\in A_{1}, \end{array}$$

(41) $$\begin{array}{ccc} \left(R_{x}S_{x}-R_{y}S_{y}\right) & & =(R_{1}^{\left(1\right)}S_{1}^{\left(1\right)}+R_{-1}^{\left(1\right)}S_{-1}^{\left(1\right)})\\ & & ={\left(R^{\left(1\right)}⊗S^{\left(1\right)}\right)}_{2}^{2}+{\left(R^{\left(1\right)}⊗S^{\left(1\right)}\right)}_{-2}^{2}\in A_{1}, \end{array}$$

(42) $$\begin{array}{ccc} \left(R_{x}S_{y}+R_{y}S_{x}\right) & & =i(R_{1}^{\left(1\right)}S_{1}^{\left(1\right)}-R_{-1}^{\left(1\right)}S_{-1}^{\left(1\right)})\\ & & =i{\left(R^{\left(1\right)}⊗S^{\left(1\right)}\right)}_{2}^{2}-i{\left(R^{\left(1\right)}⊗S^{\left(1\right)}\right)}_{-2}^{2}\in B_{2}, \end{array}$$

(43) $$\begin{array}{ccc} \left(R_{x}S_{z}+R_{z}S_{x}\right) & & =R_{0}^{\left(1\right)}\left(S_{-1}^{\left(1\right)}-S_{1}^{\left(1\right)}\right)+\left(R_{-1}^{\left(1\right)}-R_{1}^{\left(1\right)}\right)S_{0}^{\left(1\right)}\\ & & =2{\left(R^{\left(1\right)}⊗S^{\left(1\right)}\right)}_{-1}^{2}-2{\left(R^{\left(1\right)}⊗S^{\left(1\right)}\right)}_{1}^{2}\in B_{1}, \end{array}$$

and

(44) $$\begin{array}{ccc} \left(R_{y}S_{z}+R_{z}S_{y}\right) & & =R_{0}^{\left(1\right)}\left(S_{-1}^{\left(1\right)}-S_{1}^{\left(1\right)}\right)-\left(R_{-1}^{\left(1\right)}-R_{1}^{\left(1\right)}\right)S_{0}^{\left(1\right)}\\ & & =2i{\left(R^{\left(1\right)}⊗S^{\left(1\right)}\right)}_{-1}^{2}+2i{\left(R^{\left(1\right)}⊗S^{\left(1\right)}\right)}_{1}^{2}\in A_{2}. \end{array}$$

Following Equation (26) and subsequent discussion, one can expect that the symmetry γ in Equation (35) is $A_{2}$ for a parallel band (the symmetry of the states ∣v〉 and $|\tilde{v}\rangle$ is the same), or $B_{2}$ for a perpendicular band (the symmetry of the states ∣v〉 and $|\tilde{v}\rangle$ is different). We consider here both types of bands, taking into account Equations (40)–(44) and the symmetry of the $k_{Z\alpha }^{\Gamma }$ operators ($k_{Zx}^{A_{2}}$, $k_{Zy}^{B_{1}}$, or $k_{Zz}^{B_{2}}$).

### 5.1. Parallel Ro-Vibrational Bands

As was mentioned above, the index γ in Equation (35) for a parallel band is equal to $A_{2}$. This means (taking into account Equations (40)–(43)) that the operator $\frac{1}{2}{\left\{\sum_{\alpha }k_{Z\alpha }^{\Gamma },\;\mu_{\mathrm{sp}-\mathrm{rot}}^{\tilde{\Gamma }}\right\}}_{+}^{A_{2}}$ in Equation (35) should be taken as:

(45) $$\begin{array}{ccc} \frac{1}{2}{\left\{\sum_{\alpha }k_{Z\alpha }^{\Gamma },\;\mu_{\mathrm{sp}-\mathrm{rot}}^{\tilde{\Gamma }}\right\}}_{+}^{A_{2}} & & =\frac{1}{2}{\left\{k_{Zx},\;{\tilde{\mu }}_{1}^{\left(v-\tilde{v}\right)}\left(R\cdot S\right)+{\tilde{\mu }}_{2}^{\left(v-\tilde{v}\right)}\left(R_{z}S_{z}\right)+{\tilde{\mu }}_{3}^{\left(v-\tilde{v}\right)}\left(R_{x}S_{x}-R_{y}S_{y}\right)\right\}}_{+}\\ & & +\frac{1}{2}{\left\{k_{Zy},\;{\tilde{\mu }}_{4}^{\left(v-\tilde{v}\right)}\left(R_{x}S_{y}+R_{y}S_{x}\right)+\right\}}_{+}\\ & & +\frac{1}{2}{\left\{k_{Zz},\;{\tilde{\mu }}_{5}^{\left(v-\tilde{v}\right)}\left(R_{x}S_{z}+R_{z}S_{x}\right)\right\}}_{+}. \end{array}$$

Let us apply the first term of the operator, Equation (45), in the right-hand side of Equation (35). After some transformation, one can obtain the following result:

(46) $$\begin{array}{ccc} & & {\left(\langle J|⊗\langle S|\right)}_{k}^{N}\frac{1}{2}{\left\{k_{Zx},\;{\tilde{\mu }}_{1}^{\left(v-\tilde{v}\right)}\left(R\cdot S\right)\right\}}_{+}{\left(|\tilde{J}\rangle ⊗|S\rangle \right)}_{\tilde{k}}^{\tilde{N}}\\ & & =\frac{1}{2}{\tilde{\mu }}_{1}^{\left(v-\tilde{v}\right)}\sum_{J^{\prime }N^{\prime }k^{\prime }}\left\{\left[{\left(\langle J|⊗\langle S|\right)}_{k}^{N}\left\{k_{Zx}\right\}{\left(|J^{\prime }\rangle ⊗|S\rangle \right)}_{k^{\prime }}^{N^{\prime }}\right]\left[{\left(\langle J^{\prime }\left|⊗\langle S\right|\right)}_{k^{\prime }}^{N^{\prime }}\left\{(R\cdot S)\right\}{\left(|\tilde{J}\rangle ⊗|S\rangle \right)}_{\tilde{k}}^{\tilde{N}}\right]\right\}\\ & & +\frac{1}{2}{\tilde{\mu }}_{1}^{\left(v-\tilde{v}\right)}\sum_{J^{\prime }N^{\prime }k^{\prime }}\left\{\left[{\left(\langle J|⊗\langle S|\right)}_{k}^{N}\left\{(R\cdot S)\right\}{\left(|J^{\prime }\rangle ⊗|S\rangle \right)}_{k^{\prime }}^{N^{\prime }}\right]\left[{\left(\langle J^{\prime }\left|⊗\langle S\right|\right)}_{k^{\prime }}^{N^{\prime }}\left\{k_{Zx}\right\}{\left(|\tilde{J}\rangle ⊗|S\rangle \right)}_{\tilde{k}}^{\tilde{N}}\right]\right\}\\ & & =\mu_{1}^{\left(v-\tilde{v}\right)}\sqrt{\tilde{g}\left(NJ,\tilde{N}\tilde{J}\right)}\;\;\langle Jk|k_{Zx}|\tilde{J}\;\tilde{k}\rangle \left[\tilde{J}\left(\tilde{J}+1\right)-\tilde{N}\left(\tilde{N}+1\right)+J\left(J+1\right)-N\left(N+1\right)-2S\left(S+1\right)\right], \end{array}$$

where $\left(\tilde{k}=k\pm 1\right)$, and we collected all coefficients from the calculated matrix elements and the ${\tilde{\mu }}_{1}^{\left(v-\tilde{v}\right)}$-value in the new parameter $\mu_{1}^{\left(v-\tilde{v}\right)}$. Analogously, nonzero matrix elements of the second term of Equation (45) can be easily obtained, and they have the following form:

(47) $$\begin{array}{ccc} & & {\left(\langle J|⊗\langle S|\right)}_{k}^{N}\frac{1}{2}{\left\{k_{Zx},\;{\tilde{\mu }}_{2}^{\left(v-\tilde{v}\right)}\left(R_{z}S_{z}\right)\right\}}_{+}{\left(|\tilde{J}\rangle ⊗|S\rangle \right)}_{\tilde{k}}^{\tilde{N}}=\mu_{2}^{\left(v-\tilde{v}\right)}\sqrt{\tilde{g}\left(NJ,\tilde{N}\tilde{J}\right)}\;\;\langle Jk|k_{Zx}|\tilde{J}\;\tilde{k}\rangle \\ & & \times \left\{\frac{k^{2}}{N(N+1)}\left[J(J+1)-N(N+1)-S(S+1)\right]+\frac{{\tilde{k}}^{2}}{\tilde{N}\left(\tilde{N}+1\right)}\left[\tilde{J}\left(\tilde{J}+1\right)-\tilde{N}\left(\tilde{N}+1\right)-S\left(S+1\right)\right]\right\}, \end{array}$$

where, again, $\left(\tilde{k}=k\pm 1\right)$.

With regard to the three remaining terms in Equation (45), determining their matrix elements is not an easy problem. To solve it, some preliminary discussion is needed. As one can see from Equations (46) and (47), they give only corrections to the main parts, $\sqrt{\tilde{g}\left(NJ,\tilde{N}\tilde{J}\right)}\;\;\langle Jk|k_{Zx}|\tilde{J}\;\tilde{k}\rangle$ (see Equation (38)), of transitions with Δk=±1. In this case (if one takes into account Table 3), it is not difficult to see that the values of the $\tilde{g}\left(NJ,\tilde{N}\tilde{J}\right)$-coefficients with ΔJ≠ΔN are considerably smaller in comparison to values of $\tilde{g}\left(NJ,\tilde{N}\tilde{J}\right)$-coefficients with ΔJ=ΔN. Taking into account that the discussed centrifugal spin–rotational corrections themselves are small corrections to the main terms, we will not further take into account the effects that correspond in Table 3 terms with ΔJ≠ΔN. We will also take into account the evident fact that matrix elements of the operators, Equations (41) and (42), are nonzero only for Δk=±2 and ΔN=0,±1, and matrix elements of the operators, Equations (43) and (44), are nonzero only for Δk=±1 and ΔN=0,±1. Taking all of these into account, after some transformation, one can produce the following general result for the third term of Equation (45):

(48) $$\begin{array}{ccc} & & {\left(\langle J|⊗\langle S|\right)}_{k}^{N}\frac{1}{2}{\left\{k_{Zx},\;{\tilde{\mu }}_{3}^{\left(v-\tilde{v}\right)}\left(R_{x}S_{x}-R_{y}S_{y}\right)\right\}}_{+}{\left(|\tilde{J}\rangle ⊗|S\rangle \right)}_{\tilde{k}}^{\tilde{N}}\\ & & =\frac{1}{2}{\tilde{\mu }}_{3}^{\left(v-\tilde{v}\right)}\sum_{J^{\prime }N^{\prime }k^{\prime }}\left\{\left[{\left(\langle J|⊗\langle S|\right)}_{k}^{N}\left\{k_{Zx}\right\}{\left(|J^{\prime }\rangle ⊗|S\rangle \right)}_{k^{\prime }}^{N^{\prime }}\right]\left[{\left(\langle J^{\prime }\left|⊗\langle S\right|\right)}_{k^{\prime }}^{N^{\prime }}\left\{(R_{1}^{\left(1\right)}S_{1}^{\left(1\right)}+R_{-1}^{\left(1\right)}S_{-1}^{\left(1\right)})\right\}{\left(|\tilde{J}\rangle ⊗|S\rangle \right)}_{\tilde{k}}^{\tilde{N}}\right]\right\}\\ & & +\frac{1}{2}{\tilde{\mu }}_{3}^{\left(v-\tilde{v}\right)}\sum_{J^{\prime }N^{\prime }k^{\prime }}\left\{\left[{\left(\langle J|⊗\langle S|\right)}_{k}^{N}\left\{(R_{1}^{\left(1\right)}S_{1}^{\left(1\right)}+R_{-1}^{\left(1\right)}S_{-1}^{\left(1\right)})\right\}{\left(|J^{\prime }\rangle ⊗|S\rangle \right)}_{k^{\prime }}^{N^{\prime }}\right]\left[{\left(\langle J^{\prime }\left|⊗\langle S\right|\right)}_{k^{\prime }}^{N^{\prime }}\left\{k_{Zx}\right\}{\left(|\tilde{J}\rangle ⊗|S\rangle \right)}_{\tilde{k}}^{\tilde{N}}\right]\right\}\\ & & =\mu_{3}^{\left(v-\tilde{v}\right)}\sqrt{\tilde{g}\left(NJ,L\tilde{J}\right)}\;\;\langle Jk|k_{Zx}|\tilde{J}\;l\rangle \left\{{\left(\langle \tilde{J}\left|⊗\langle S\right|\right)}_{l}^{L}\left[{\left(R^{\left(1\right)}⊗S^{\left(1\right)}\right)}_{2}^{2}+{\left(R^{\left(1\right)}⊗S^{\left(1\right)}\right)}_{-2}^{2}\right]{\left(|\tilde{J}\rangle ⊗|S\rangle \right)}_{\tilde{k}}^{\tilde{N}}\right\}\\ & & +\mu_{3}^{\left(v-\tilde{v}\right)}\left\{{\left(\langle J|⊗\langle S|\right)}_{k}^{N}\left[{\left(R^{\left(1\right)}⊗S^{\left(1\right)}\right)}_{2}^{2}+{\left(R^{\left(1\right)}⊗S^{\left(1\right)}\right)}_{-2}^{2}\right]{\left(|J\rangle ⊗|S\rangle \right)}_{m}^{M}\right\}\sqrt{\tilde{g}\left(MJ,\tilde{N}\tilde{J}\right)}\;\;\langle Jm|k_{Zx}|\tilde{J}\;\tilde{k}\rangle . \end{array}$$

One can see that, in the final result, the initial summation is absent; the indexes in Equation (48) are: $\tilde{k}=k\pm 3$, or $\tilde{k}=k\pm 1$; $\tilde{N}=N+\Delta N$ (ΔN=0,±1,±2); $\tilde{J}=J+\Delta J$ (ΔJ=0,±1); L=N+ΔJ, $M=\tilde{N}-\Delta J$. Possible combinations of indexes for nonzero values of matrix elements are shown in Table 4, and nonzero matrix elements of the ${\left(R^{1}⊗S^{1}\right)}_{\pm 2}^{2}$ operators are:

(49) $$\begin{array}{ccc} & & {\left(\langle J|⊗\langle S|\right)}_{k}^{N}{\left(R^{\left(1\right)}⊗S^{\left(1\right)}\right)}_{\pm 2}^{2}{\left(|J\rangle ⊗|S\rangle \right)}_{\tilde{k}=k\mp 2}^{N}\\ & & ={\left(-1\right)}^{2(N-J)}\frac{\left(2N+1\right)}{4J(2J+1)}{\left\{(N\pm k-1)(N\pm k)(N\mp k+1)(N\mp k+2)\right\}}^{1/2}, \end{array}$$

and

(50) $$\begin{array}{ccc} & & {\left(\langle J|⊗\langle S|\right)}_{k}^{N}{\left(R^{\left(1\right)}⊗S^{\left(1\right)}\right)}_{\pm 2}^{2}{\left(|J\rangle ⊗|S\rangle \right)}_{\tilde{k}=k\mp 2}^{\left(N+\Delta N\right)}\\ & & =\frac{\Delta N(k-\tilde{k})}{4}{\left\{\frac{\left(N\pm k)(N\mp k+1)[(N+1)+\Delta N(2\mp k)][N+\Delta N(2\mp k)\right]}{\left(2J+1)(2J+2+\Delta N\right)}\right\}}^{1/2}. \end{array}$$

As one can see from the comparison of the right-hand sides of Equations (49) and (50), all of them are values of the same order and are approximately proportional to *N*. Taking into account that, firstly, the $\mu_{3}^{\left(v-\tilde{v}\right)}$ in Equation (48) is a small parameter in comparison with the main $\mu_{1}^{\left(v-\tilde{v}\right)}$ one, and, secondly, in Equation (48), $\left(N-M\right)=\left(J-\tilde{J}\right)$ (see discussion above), one can conclude that an influence of the terms, Equation (48), on the absolute line strengths of the molecule considered is of the same order of value as the influence of the main $\mu_{1}^{\left(v-\tilde{v}\right)}\sqrt{\tilde{g}\left(NJ,M\tilde{J}\right)}\;\;\langle Jk|k_{Zx}|\tilde{J}\;k\rangle$ parts for the condition $\left(N-M\right)\neq \left(J-\tilde{J}\right)$ (“forbidden” transitions). If speaking about “allowed” transitions $\left(N-M\right)=\left(J-\tilde{J}\right)$, Equation (48) gives only small corrections to the main terms (which nevertheless can increase significantly with an increasing value of quantum number *N*). At the same time, for “forbidden” transitions, Equation (48) gives results that are comparable by order of value with such “main” parts. Moreover (as is seen from Table 3), the “main” parts of “forbidden” transitions decrease by $1/N^{2}$ with the increasing quantum number *N* while the values, Equation (48), increase by *N*. This can be considered as an important consequence of the obtained result. One more interesting consequence is the fact that Equation (48) allows for transitions with the value Δk=±,3 or ΔN=±2, which are absent in the description by formulas that use data from Table 3. The same as for the “forbidden” transitions with Δk=±1 and ΔN=0,±1, corresponding values for ”allowed“ transitions with Δk=±3 or ΔN=±2 increase by *N* with increasing *N*.

The analogous consideration for the fourth term in Equation (45) leads to the following result:

(51) $$\begin{array}{ccc} & & {\left(\langle J|⊗\langle S|\right)}_{k}^{N}\frac{1}{2}{\left\{k_{Zy},\;{\tilde{\mu }}_{4}^{\left(v-\tilde{v}\right)}\left(R_{x}S_{y}+R_{y}S_{x}\right)\right\}}_{+}{\left(|\tilde{J}\rangle ⊗|S\rangle \right)}_{\tilde{k}}^{\tilde{N}}=\\ & & =\mu_{4}^{\left(v-\tilde{v}\right)}\sqrt{\tilde{g}\left(NJ,M\tilde{J}\right)}\;\;\langle Jk|k_{Zx}|\tilde{J}\;l\rangle \left(l-k\right)\left\{{\left(\langle \tilde{J}\left|⊗\langle S\right|\right)}_{l}^{M}\left[{\left(R^{\left(1\right)}⊗S^{\left(1\right)}\right)}_{2}^{2}-{\left(R^{\left(1\right)}⊗S^{\left(1\right)}\right)}_{-2}^{2}\right]{\left(|\tilde{J}\rangle ⊗|S\rangle \right)}_{\tilde{k}}^{\tilde{N}}\right\}\\ & & +\mu_{4}^{\left(v-\tilde{v}\right)}\sqrt{\tilde{g}\left(NJ,M\tilde{J}\right)}\;\;\langle Jk|k_{Zx}|\tilde{J}\;l\rangle \left(l-k\right)\left\{{\left(\langle \tilde{J}\left|⊗\langle S\right|\right)}_{l}^{M}\left[{\left(R^{\left(1\right)}⊗S^{\left(1\right)}\right)}_{2}^{2}-{\left(R^{\left(1\right)}⊗S^{\left(1\right)}\right)}_{-2}^{2}\right]{\left(|\tilde{J}\rangle ⊗|S\rangle \right)}_{\tilde{k}}^{\tilde{N}}\right\}, \end{array}$$

if one takes into account the known relation (see, e.g., [43]):

(52) $$\begin{array}{c} \langle Jk|ik_{Zy}|\tilde{J}\;l\rangle =\left(l-k\right)\langle Jk|k_{Zx}|\tilde{J}\;l\rangle . \end{array}$$

And, finally, for the fifth term of Equation (45), it is possible to obtain the following relation:

(53) $$\begin{array}{ccc} & & {\left(\langle J|⊗\langle S|\right)}_{k}^{N}\frac{1}{2}{\left\{k_{Zx},\;{\tilde{\mu }}_{5}^{\left(v-\tilde{v}\right)}\left(R_{x}S_{z}+R_{z}S_{x}\right)\right\}}_{+}{\left(|\tilde{J}\rangle ⊗|S\rangle \right)}_{\tilde{k}}^{\tilde{N}}\\ & & =\mu_{5}^{\left(v-\tilde{v}\right)}\sqrt{\tilde{g}\left(NJ,L\tilde{J}\right)}\;\;\langle Jk|k_{Zx}|\tilde{J}\;l\rangle \left\{{\left(\langle \tilde{J}\left|⊗\langle S\right|\right)}_{l}^{L}\left[{\left(R^{\left(1\right)}⊗S^{\left(1\right)}\right)}_{-1}^{2}-{\left(R^{\left(1\right)}⊗S^{\left(1\right)}\right)}_{1}^{2}\right]{\left(|\tilde{J}\rangle ⊗|S\rangle \right)}_{\tilde{k}}^{\tilde{N}}\right\}\\ & & +\mu_{5}^{\left(v-\tilde{v}\right)}\left\{{\left(\langle J|⊗\langle S|\right)}_{k}^{N}\left[{\left(R^{\left(1\right)}⊗S^{1}\right)}_{-1}^{2}-{\left(R^{\left(1\right)}⊗S^{\left(1\right)}\right)}_{1}^{2}\right]{\left(|J\rangle ⊗|S\rangle \right)}_{m}^{M}\right\}\sqrt{\tilde{g}\left(MJ,\tilde{N}\tilde{J}\right)}\;\;\langle Jm|k_{Zx}|\tilde{J}\;\tilde{k}\rangle , \end{array}$$

where evidently weak “forbidden” transitions with Δk=0,±2 are described. Indexes in Equation (53) are: $\tilde{k}=k$, or $\tilde{k}=k\pm 2$; $\tilde{N}=N+\Delta N$ (ΔN=0,±1,±2); $\tilde{J}=J+\Delta J$ (ΔJ=0,±1); L=N+ΔJ, $M=\tilde{N}-\Delta J$, and nonzero matrix elements of the ${\left(R^{\left(1\right)}⊗S^{\left(1\right)}\right)}_{\pm 1}^{2}$ operators are:

(54) $$\begin{array}{ccc} & & {\left(\langle J|⊗\langle S|\right)}_{k}^{N}{\left(R^{\left(1\right)}⊗S^{\left(1\right)}\right)}_{\pm 1}^{2}{\left(|J\rangle ⊗|S\rangle \right)}_{\tilde{k}=k\mp 1}^{N}\\ & & ={\left(-1\right)}^{2(N-J)}\frac{\left(2N+1)(1\mp 2k\right)}{4J(2J+1)}{\left\{(N\pm k)(N\mp k+1)\right\}}^{1/2} \end{array}$$

and

(55) $$\begin{array}{ccc} & & {\left(\langle J|⊗\langle S|\right)}_{k}^{N}{\left(R^{\left(1\right)}⊗S^{\left(1\right)}\right)}_{\pm 1}^{2}{\left(|J\rangle ⊗|S\rangle \right)}_{\tilde{k}=k\mp 1}^{\left(N+\Delta N\right)}\\ & & =\frac{\Delta N\left(k-\tilde{k}\right)\left(N\mp 2k+1\right)}{4}{\left\{\frac{\left(N\mp k+1)[N+\Delta N(2\mp k)\right]}{\left(2J+1)(2J+2+\Delta N\right)}\right\}}^{1/2}. \end{array}$$

### 5.2. Perpendicular Ro-Vibrational Bands

As discussed above, for a perpendicular ro-vibrational band, the index γ in Equation (35) is equal to $B_{2}$. This means that the analogous form of Equation (45) for a perpendicular band should be written as

(56) $$\begin{array}{ccc} \frac{1}{2}{\left\{\sum_{\alpha }k_{Z\alpha }^{\Gamma },\;\mu_{\mathrm{sp}-\mathrm{rot}}^{\tilde{\Gamma }}\right\}}_{+}^{B_{2}} & & =\frac{1}{2}{\left\{k_{Zz},\;{\tilde{\mu }}_{1}^{\left(v-\tilde{v}\right)}\left(R\cdot S\right)+{\tilde{\mu }}_{2}^{\left(v-\tilde{v}\right)}\left(R_{z}S_{z}\right)+{\tilde{\mu }}_{3}^{\left(v-\tilde{v}\right)}\left(R_{x}S_{x}-R_{y}S_{y}\right)\right\}}_{+}\\ & & +\frac{1}{2}{\left\{k_{Zy},\;{\tilde{\mu }}_{4}^{\left(v-\tilde{v}\right)}\left[(R_{y}S_{z}+R_{z}S_{y})\right]\right\}}_{+}\\ & & +\frac{1}{2}{\left\{k_{Zx},\;{\tilde{\mu }}_{5}^{\left(v-\tilde{v}\right)}\left[(R_{x}S_{z}+R_{z}S_{x})\right]\right\}}_{+}. \end{array}$$

In the same way as in Section 5.1, it is possible to show that, for the first two operators in Equation (56), the corresponding matrix elements have the same form as Equations (46) and (47) if one changes the values $\langle Jk|k_{Zx}|\tilde{J}\;\tilde{k}\rangle$ in Equations (46) and (47) by the $\langle Jk|k_{Zz}|\tilde{J}\;\tilde{k}\rangle$ ones:

(57) $$\begin{array}{ccc} & & {\left(\langle J|⊗\langle S|\right)}_{k}^{N}\frac{1}{2}{\left\{k_{Zz},\;{\tilde{\mu }}_{1}^{\left(v-\tilde{v}\right)}\left(R\cdot S\right)\right\}}_{+}{\left(|\tilde{J}\rangle ⊗|S\rangle \right)}_{\tilde{k}}^{\tilde{N}}\\ & & =\mu_{1}^{\left(v-\tilde{v}\right)}\sqrt{\tilde{g}\left(NJ,\tilde{N}\tilde{J}\right)}\;\;\langle Jk|k_{Zz}|\tilde{J}\;\tilde{k}\rangle \left[\tilde{J}\left(\tilde{J}+1\right)-\tilde{N}\left(\tilde{N}+1\right)+J\left(J+1\right)-N\left(N+1\right)-2S\left(S+1\right)\right] \end{array}$$

and

(58) $$\begin{array}{ccc} & & {\left(\langle J|⊗\langle S|\right)}_{k}^{N}\frac{1}{2}{\left\{k_{Zz},\;{\tilde{\mu }}_{2}^{\left(v-\tilde{v}\right)}\left(R_{z}S_{z}\right)\right\}}_{+}{\left(|\tilde{J}\rangle ⊗|S\rangle \right)}_{\tilde{k}}^{\tilde{N}}=\mu_{2}^{\left(v-\tilde{v}\right)}\sqrt{\tilde{g}\left(NJ,\tilde{N}\tilde{J}\right)}\;\;\langle Jk|k_{Zz}|\tilde{J}\;\tilde{k}\rangle \\ & & \times \left\{\frac{k^{2}}{N(N+1)}\left[J(J+1)-N(N+1)-S(S+1)\right]+\frac{{\tilde{k}}^{2}}{\tilde{N}\left(\tilde{N}+1\right)}\left[\tilde{J}\left(\tilde{J}+1\right)-\tilde{N}\left(\tilde{N}+1\right)-S\left(S+1\right)\right]\right\}. \end{array}$$

Here, of course, one should take into account that $\left(\tilde{k}=k\right)$.

For the third term in Equation (56), the use of the scheme from Section 5.1 gives the following result:

(59) $$\begin{array}{ccc} & & {\left(\langle J|⊗\langle S|\right)}_{k}^{N}\frac{1}{2}{\left\{k_{Zz},\;{\tilde{\mu }}_{3}^{\left(v-\tilde{v}\right)}\left(R_{x}S_{x}-R_{y}S_{y}\right)\right\}}_{+}{\left(|\tilde{J}\rangle ⊗|S\rangle \right)}_{\tilde{k}}^{\tilde{N}}\\ & & =\mu_{3}^{\left(v-\tilde{v}\right)}\sqrt{\tilde{g}\left(NJ,L\tilde{J}\right)}\;\;\langle Jk|k_{Zz}|\tilde{J}\;k\rangle \left\{{\left(\langle \tilde{J}\left|⊗\langle S\right|\right)}_{l=k}^{L}\left[{\left(R^{\left(1\right)}⊗S^{\left(1\right)}\right)}_{2}^{2}+{\left(R^{\left(1\right)}⊗S^{\left(1\right)}\right)}_{-2}^{2}\right]{\left(|\tilde{J}\rangle ⊗|S\rangle \right)}_{\tilde{k}}^{\tilde{N}}\right\}\\ & & +\mu_{3}^{\left(v-\tilde{v}\right)}\left\{{\left(\langle J|⊗\langle S|\right)}_{k}^{N}\left[{\left(R^{\left(1\right)}⊗S^{\left(1\right)}\right)}_{2}^{2}+{\left(R^{\left(1\right)}⊗S^{\left(1\right)}\right)}_{-2}^{2}\right]{\left(|J\rangle ⊗|S\rangle \right)}_{m=\tilde{k}}^{M}\right\}\sqrt{\tilde{g}\left(MJ,\tilde{N}\tilde{J}\right)}\;\;\langle J\tilde{k}|k_{Zz}|\tilde{J}\;\tilde{k}\rangle , \end{array}$$

where $\tilde{k}=k\pm 2$, $\tilde{N}=N+\Delta N$ (ΔN=0,±1,±2); $\tilde{J}=J+\Delta J$ (ΔJ=0,±1); L=N+ΔJ, $M=\tilde{N}-\Delta J$. Possible combinations of indexes for nonzero values of matrix elements are the same as in Table 4 (in this case, one should not take into account the footnote to Table 4; possible values of indexes *l* and *m* are given directly in Equation (59)), and nonzero matrix elements of the ${\left(R^{\left(1\right)}⊗S^{\left(1\right)}\right)}_{\pm 2}^{2}$ operators are presented in Equations (49) and (50).

It is not difficult to show that the nonzero matrix elements of the fourth and fifth term of Equation (56) are:

(60) $$\begin{array}{ccc} & & {\left(\langle J|⊗\langle S|\right)}_{k}^{N}\frac{1}{2}{\left\{k_{Zy},\;{\tilde{\mu }}_{4}^{\left(v-\tilde{v}\right)}\left(R_{y}S_{z}+R_{z}S_{y}\right)\right\}}_{+}{\left(|\tilde{J}\rangle ⊗|S\rangle \right)}_{\tilde{k}}^{\tilde{N}}\\ & & =\mu_{4}^{\left(v-\tilde{v}\right)}\left(l-k\right)\sqrt{\tilde{g}\left(NJ,L\tilde{J}\right)}\;\;\langle Jk|k_{Zx}|\tilde{J}\;l\rangle \left\{{\left(\langle \tilde{J}\left|⊗\langle S\right|\right)}_{l}^{L}\left[{\left(R^{\left(1\right)}⊗S^{\left(1\right)}\right)}_{-1}^{2}+{\left(R^{\left(1\right)}⊗S^{\left(1\right)}\right)}_{1}^{2}\right]{\left(|\tilde{J}\rangle ⊗|S\rangle \right)}_{\tilde{k}}^{\tilde{N}}\right\}\\ & & +\mu_{4}^{\left(v-\tilde{v}\right)}\left(\tilde{k}-m\right)\left\{{\left(\langle J|⊗\langle S|\right)}_{k}^{N}\left[{\left(R^{\left(1\right)}⊗S^{\left(1\right)}\right)}_{-1}^{2}+{\left(R^{\left(1\right)}⊗S^{\left(1\right)}\right)}_{1}^{2}\right]{\left(|J\rangle ⊗|S\rangle \right)}_{m}^{M}\right\}\sqrt{\tilde{g}\left(MJ,\tilde{N}\tilde{J}\right)}\;\;\langle Jm|k_{Zx}|\tilde{J}\;\tilde{k}\rangle \end{array}$$

(here, we took into account Equation (53)), and

(61) $$\begin{array}{ccc} & & {\left(\langle J|⊗\langle S|\right)}_{k}^{N}\frac{1}{2}{\left\{k_{Zy},\;{\tilde{\mu }}_{5}^{\left(v-\tilde{v}\right)}\left(R_{x}S_{z}+R_{z}S_{x}\right)\right\}}_{+}{\left(|\tilde{J}\rangle ⊗|S\rangle \right)}_{\tilde{k}}^{\tilde{N}}\\ & & =\mu_{5}^{\left(v-\tilde{v}\right)}\sqrt{\tilde{g}\left(NJ,L\tilde{J}\right)}\;\;\langle Jk|k_{Zy}|\tilde{J}\;l\rangle \left\{{\left(\langle \tilde{J}\left|⊗\langle S\right|\right)}_{l}^{L}\left[{\left(R^{\left(1\right)}⊗S^{\left(1\right)}\right)}_{-1}^{2}-{\left(R^{\left(1\right)}⊗S^{\left(1\right)}\right)}_{1}^{2}\right]{\left(|\tilde{J}\rangle ⊗|S\rangle \right)}_{\tilde{k}}^{\tilde{N}}\right\}\\ & & +\mu_{5}^{\left(v-\tilde{v}\right)}\left\{{\left(\langle J|⊗\langle S|\right)}_{k}^{N}\left[{\left(R^{\left(1\right)}⊗S^{\left(1\right)}\right)}_{-1}^{2}-{\left(R^{\left(1\right)}⊗S^{\left(1\right)}\right)}_{1}^{2}\right]{\left(|J\rangle ⊗|S\rangle \right)}_{m}^{M}\right\}\sqrt{\tilde{g}\left(MJ,\tilde{N}\tilde{J}\right)}\;\;\langle Jm|k_{Zy}|\tilde{J}\;\tilde{k}\rangle . \end{array}$$

The values of the indexes $\tilde{N},\tilde{J},L,M$ in Equations (60) and (61) are the same as in Equations (48), (53), and (59); $\tilde{k}=k$ or ±k; l=k±1; m=k±1; and nonzero matrix elements of the ${\left(R^{\left(1\right)}⊗S^{\left(1\right)}\right)}_{\pm 1}^{2}$ operators can be taken from Equations (54) and (55).

As an illustration of the importance and correctness of the results, let us consider one of the obtained formulas, e.g., Equation (59), which is applied to perpendicular spin–ro-vibrational bands with the allowed transitions ΔK=0. In accordance with this formula, “forbidden” transitions with ΔK=±2 can also be seen in absorption spectra of the considered type of molecules. If one uses the matrix elements (49) and (50) in Equation (59) and takes into account Equations (13), (15), and (17), then it is possible to conclude the following: the matrix elements which correspond to the *Q* that transitions decrease by $\frac{k}{\sqrt{N}}$ with increasing quantum number *N*, and matrix elements that correspond to the *P* and *R* transitions increase by $\left(\frac{N^{2}-k^{2}}{\sqrt{N}}\right)$ with increasing *N*. For this reason, we present here, as an illustration, a set of *R* transitions of the $\left(N+1\;K_{a}=0\;K_{c}=N\;\left(\pm \right)\right)⟵\left(N\;K_{a}=2\;K_{c}=\left(N-2\right)\;\left(\pm \right)\right)$−type for the $\nu_{3}$ band of the ^35^ClO_2_ molecule whose experimental values can be found in Ref. [36] (they are reproduced from [36] in column 3 of Table 5). Column 2 of Table 5 presents theoretically predicted values of the same transition frequencies. These predicted values were obtained as differences between values of corresponding spin–ro-vibrational energies of the (001) upper vibrational state (the latter have been taken from Table 4 of Ref. [36]) and those of the ground vibrational state (in this case, spin–rotational energies of the ground vibrational state have been calculated with the parameters from column 2 of Table 2; [36]).

**Figure 1:**
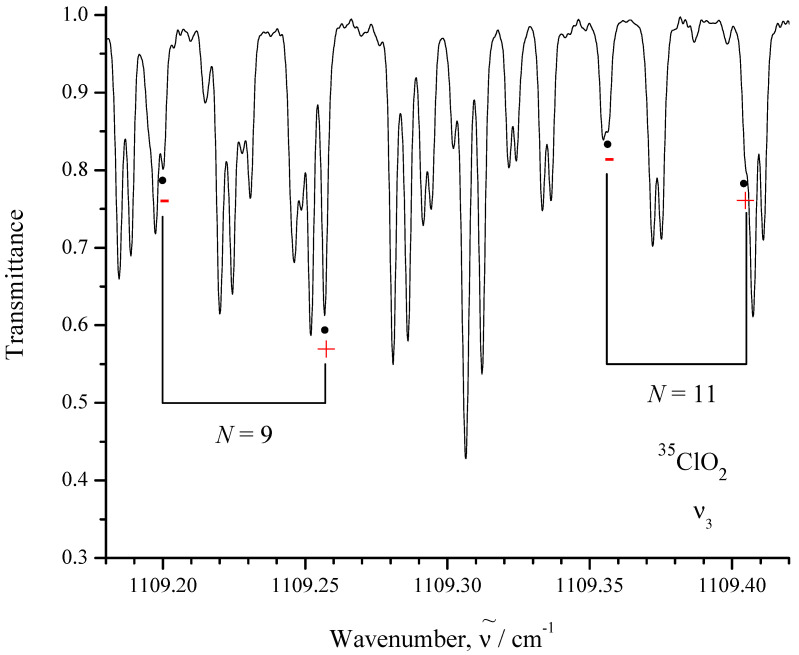
Small portion of the experimental spectrum of ClO_2_ compared with Ref. [36]. “Forbidden transitions” ΔK=±2 are marked by dark circles. Experimental conditions: resolution is 0.0015 cm^−1^; number of scans is 400; source is a Globar; detector is a MCT313; beam-splitter is made from KBr; optical path length is 0.23 m; aperture is 1.15 mm; temperature is 22 ± 0.3 °C; pressure is 100 Pa; calibration was performed by CO_2_ and H_2_O spectral lines.

Column 1 of this table indicates quantum numbers of the upper and lower spin–ro-vibrational states of a transition (in this case, sign (+) corresponds to the value J=N+1/2 and sign (−) corresponds to the value J=N−1/2). Columns 2 and 3 present calculated line positions (in cm^−1^) and corresponding experimental line positions from spectrum I of Ref. [36] (also in cm^−1^). The values in column 4 are transmittances of experimental lines. One can see that the “forbidden” transition is strong enough (for a comparison with the “allowed” transitions of the $\nu_{3}$ band, see the small fragment of the mentioned experimental spectrum in Figure 1). One can argue that the reason for the appearance of the discussed transitions can be a superposition of the spin–rotational basic functions with ΔK=0 and ΔK=±2 in the effective Hamiltonian eigenfunctions, which are used in the calculation of matrix elements of the effective dipole moment of a molecule. However, the analysis of corresponding wave functions and the estimation of corresponding numerical values show that such influence of superpositions in wave functions is negligible in comparison with the effect of Equation (59).

## 6. Conclusions

We derived a new model of the effective dipole moment of the XY_2_ (C_2*v*_-symmetry) molecule in a doublet electronic state by taking into account spin–rotational centrifugal corrections that have never been considered earlier for such kind of problems. Corresponding relations (which are necessary for determination of absolute spin–ro-vibrational transition strengths and which contain all effects known up to now, as special cases) are obtained on the basis of the irreducible tensorial sets theory. The derived results allow us to take into account both the higher-order corrections to the allowed transitions and also to describe weak transitions of the ΔK=±2,±3−types in the parallel bands, of the ΔK=±1,±2−types in the perpendicular bands, and of the ΔN=±2−type in both kinds of spin–ro-vibrational bands. To illustrate the correctness and efficiency of the derived model, we compared the estimated line strengths of a set of the “forbidden” $\Delta K_{a}=2$ transitions of the $\nu_{3}$ band of the OClO free radical with corresponding experimental data, which confirm the validity of the obtained results.

## Figures and Tables

**Table 1:** Operators and matrix elements for the perpendicular band (Δk=±1) (reproduced from Ref. [1]).

*j*	${}^{v}A_{j}$	*n*	$<\mathrm{JK}|{}^{v}A_{j}|J+\Delta J\;\;K+n\Delta K>$; ΔK=±1	
1	$\varphi_{x}$	1	$<JK|\varphi_{x}|J+\Delta J\;\;K+\Delta K>$	ΔJ=0,±1
2	$\left\{\varphi_{x},J^{2}\right\}$	1	$\left[J\left(J+1\right)+\left(J+\Delta J\right)\left(J+\Delta J+1\right)\right]<JK|\varphi_{x}|J+\Delta J\;\;K+\Delta K>$	ΔJ=0,±1
3	$\left\{\varphi_{x},J_{z}^{2}\right\}$	1	$\left[K^{2}+{\left(K+\Delta K\right)}^{2}\right]<JK\left|\varphi_{x}\right|J+\Delta J\;\;K+\Delta K>$	ΔJ=0,±1
4	$\left\{i\varphi_{y},J_{z}\right\}$	1	$\left(1+2K\Delta K\right)<JK|\varphi_{x}|J+\Delta J\;\;K+\Delta K>$	ΔJ=0,±1
5	$\left\{\varphi_{z},iJ_{y}\right\}$	1	$\left(1+2K\Delta K\right)<JK|\varphi_{x}|J\;\;K+\Delta K>$	ΔJ=0
			$\left(1+2K\Delta K-2m\right)<JK|\varphi_{x}|J+\Delta J\;\;K+\Delta K>$	ΔJ=±1
6	$\left\{\varphi_{z},J_{x}J_{z}+J_{z}J_{x}\right\}$	1	${\left(1+2K\Delta K\right)}^{2}<JK\left|\varphi_{x}\right|J\;\;K+\Delta K>$	ΔJ=0
			$\left(1+2K\Delta K\right)\left(1+2K\Delta K-2m\right)<JK|\varphi_{x}|J+\Delta J\;\;K+\Delta K>$	ΔJ=±1
7	$\frac{1}{2}\left[\left\{\varphi_{x},J_{xy}^{2}\right\}-\left\{i\varphi_{y},i\left(J_{x}J_{y}+J_{y}J_{x}\right)\right\}\right]$	1	$\left[J\left(J+1\right)-K\Delta K-K^{2}-1\right]<JK\left|\varphi_{x}\right|J\;\;K+\Delta K>$	ΔJ=0
			$-\left[m\left(m-1\right)-\left(2m-1\right)K\Delta K+K^{2}+1\right]<JK\left|\varphi_{x}\right|J+\Delta J\;\;K+\Delta K>$	ΔJ=±1
	$\frac{1}{2}\left[\left\{\varphi_{x},J_{xy}^{2}\right\}+\left\{i\varphi_{y},i\left(J_{x}J_{y}+J_{y}J_{x}\right)\right\}\right]$		${\left[\left(J-K\Delta K-1\right)\left(J-K\Delta K-2\right)\left(J+K\Delta K+2\right)\left(J+K\Delta K+3\right)\right]}^{1/2}$	
8		3	$<JK|\varphi_{x}|J\;\;K+\Delta K>$	ΔJ=0
			${\left[\left(m-K\Delta K-1\right)\left(m-K\Delta K-2\right)\left(m+K\Delta K+2\right)\left(m+K\Delta K+3\right)\right]}^{1/2}$	
			$<JK|\varphi_{x}|J+\Delta J\;\;K+\Delta K>$	ΔJ=±1

**Table 2:** Operators and matrix elements for the parallel band (Δk=0) (reproduced from Ref. [1]).

*j*	${}^{v}A_{j}$	*n*	$<\mathrm{JK}|{}^{v}A_{j}|J+\Delta J\;\;K+n\Delta K>$; ΔK=±1	
1	$\varphi_{z}$	0	$<JK|\varphi_{z}|J+\Delta J\;\;K>$	ΔJ=0,±1
2	$\left\{\varphi_{z},J^{2}\right\}$	0	$\left[J\left(J+1\right)+\left(J+\Delta J\right)\left(J+\Delta J+1\right)\right]<JK|\varphi_{z}|J+\Delta J\;\;K>$	ΔJ=0,±1
3	$\left\{\varphi_{z},J_{z}^{2}\right\}$	0	$2K^{2}<JK\left|\varphi_{z}\right|J+\Delta J\;\;K>$	ΔJ=0,±1
4	$\frac{1}{2}\left[\left\{\varphi_{x},iJ_{y}\right\}-\left\{i\varphi_{y},J_{x}\right\}\right]$	0	0	ΔJ=0
		0	$m<JK|\varphi_{z}|J+\Delta J\;\;K>$	ΔJ=±1
5	$\frac{1}{2}\left\{\varphi_{x},J_{x}J_{z}+J_{z}J_{x}\right\}$	0	$\left[2\left(J\left(J+1\right)-K^{2}\right)-1\right]<JK\left|\varphi_{z}\right|J\;\;K>$	ΔJ=0
	$-\frac{1}{2}\left\{i\varphi_{y},i\left(J_{y}J_{z}+J_{z}J_{y}\right)\right\}$		$-\left(1+2K^{2}\right)<JK\left|\varphi_{z}\right|J+\Delta J\;\;K>$	ΔJ=±1
6	$\frac{1}{2}\left[\left\{\varphi_{x},iJ_{y}\right\}+\left\{i\varphi_{y},J_{x}\right\}\right]$	2	$\Delta K{\left[\left(J-K\Delta K-1\right)\left(J+K\Delta K+2\right)\right]}^{1/2}<JK\left|\varphi_{x}\right|J\;\;K+\Delta K>$	ΔJ=0
			$\Delta K{\left[\left(m-K\Delta K-1\right)\left(m+K\Delta K+2\right)\right]}^{1/2}<JK\left|\varphi_{x}\right|J+\Delta J\;\;K+\Delta K>$	ΔJ=±1
7	$\frac{1}{2}\left\{\varphi_{x},J_{x}J_{z}+J_{z}J_{x}\right\}$	2	$2\left(K+\Delta K\right){\left[\left(J-K\Delta K-1\right)\left(J+K\Delta K+2\right)\right]}^{1/2}<JK\left|\varphi_{x}\right|J\;\;K+\Delta K>$	ΔJ=0
	$+\frac{1}{2}\left\{i\varphi_{y},i\left(J_{y}J_{z}+J_{z}J_{y}\right)\right\}$		$2\left(K+\Delta K\right){\left[\left(m-K\Delta K-1\right)\left(m+K\Delta K+2\right)\right]}^{1/2}<JK\left|\varphi_{x}\right|J+\Delta J\;\;K+\Delta K>$	ΔJ=±1
8	$\left\{\varphi_{z},J_{xy}^{2}\right\}$	2	$2\left(K+\Delta K\right){\left[\left(J-K\Delta K-1\right)\left(J+K\Delta K+2\right)\right]}^{1/2}<JK\left|\varphi_{x}\right|J\;\;K+\Delta K>$	ΔJ=0
			$-2\Delta K\left(m-1-K\Delta K\right){\left[\left(m-K\Delta K-1\right)\left(m+K\Delta K+2\right)\right]}^{1/2}$	ΔJ=±1
			$<JK|\varphi_{x}|J+\Delta J\;\;K+\Delta K>$	

**Table 3:** Nonzero values of the $\tilde{g}\left(NJ,\tilde{N}\tilde{J}\right)$—coefficients (“relative intensities”) of spin–rotational components of rotational transitions.

$\tilde{N}$	$\tilde{J}$	*J*	$\Delta J=\tilde{J}-J$	Value
$\tilde{N}=N-1$	$\tilde{J}$ = $\tilde{N}+1/2$ = N−1/2	*J* = N+1/2	ΔJ=ΔN	$\frac{2N-1}{4N}$
	$\tilde{J}$ = $\tilde{N}-1/2$ = N−3/2	*J* = N−1/2	ΔJ=ΔN	$\frac{2N+1}{4N}$
	$\tilde{J}$ = $\tilde{N}+1/2$ = N−1/2	*J* = N−1/2	ΔJ≠ΔN	$\frac{1}{4N^{2}}$
	$\tilde{J}$ = $\tilde{N}-1/2$ = N−3/2	*J* = N+1/2	ΔJ≠ΔN	0
$\tilde{N}=N$	$\tilde{J}$ = N+1/2	*J* = N+1/2	ΔJ=ΔN	$\frac{N(2N+3)}{4{\left(N+1\right)}^{2}}$
	$\tilde{J}$ = N−1/2	*J* = N−1/2	ΔJ=ΔN	$\frac{\left(N+1)(2N-1\right)}{4N^{2}}$
	$\tilde{J}$ = N−1/2	*J* = N+1/2	ΔJ≠ΔN	$\frac{1}{4N(N+1)}$
	$\tilde{J}$ = N+1/2	*J* = N−1/2	ΔJ≠ΔN	$\frac{1}{4N(N+1)}$
$\tilde{N}=N+1$	$\tilde{J}$ = $\tilde{N}+1/2$ = N+3/2	*J* = N+1/2	ΔJ=ΔN	$\frac{2N+1}{4(N+1)}$
	$\tilde{J}$ = $\tilde{N}-1/2$ = N+1/2	*J* = N−1/2	ΔJ=ΔN	$\frac{2N+3}{4(N+1)}$
	$\tilde{J}$ = $\tilde{N}-1/2$ = N+1/2	*J* = N+1/2	ΔJ≠ΔN	$\frac{1}{4{\left(N+1\right)}^{2}}$
	$\tilde{J}$ = $\tilde{N}+1/2$ = N+3/2	*J* = N−1/2	ΔJ≠ΔN	0

**Table 4:** Possible combinations of indexes for nonzero values of matrix elements; Equations (48) and (52) ${}^{\left(a\right)}$.

$\Delta N=\tilde{N}-N$	*J*	$\Delta J=\tilde{J}-J$	*L*	*M*
0	N±1/2	0	*N*	*N*
	N−1/2	1	N+1	N−1
	N+1/2	−1	N−1	N+1
1	N+1/2	0	*N*	N+1
	N±1/2	1	N+1	*N*
−1	N−1/2	0	*N*	N−1
	N±1/2	−1	N−1	*N*
2	N+1/2	1	N+1	N+1
−2	N−1/2	-1	N−1	N−1

${}^{\left(a\right)}$ Only four combinations of Δl=l−k and Δm=m−k are available: for Δk=+3, Δl=+1 and Δm+2; for Δk=+1, Δl=−1 and Δm+2; for Δk=−1, Δl=+1 and Δm−2; and for Δk=−3, Δl=−1 and Δm−2.

**Table 5:** Illustration of the “forbidden” ΔK=2 transitions in the $\nu_{3}$ band of ^35^ClO_2_.

Transition			$\nu_{\mathrm{calc}.}$	$\nu_{\exp.}$	Transmitt.
$\left[N\prime =N+1\;K_{a}^{\prime }=0\;K_{c}^{\prime }\;\;\left(\sigma^{\prime }\right)\right]$	−	** $\left[N\;K_{a}=2\;K_{c}\;\;\left(\sigma \right)\right]$ **	**in cm^−1^**	**in cm^−1^**	**in %**
	**1**		**2**	**3**	**4**
$\left[4\;0\;4\;(-)\right]$	−	$\left[3\;2\;1\;(-)\right]$	1106.7267	1106.7262	92
$\left[4\;0\;4\;(+)\right]$	−	$\left[3\;2\;1\;(+)\right]$	1106.7824	1106.7828	92
$\left[6\;0\;6\;(-)\right]$	−	$\left[5\;2\;3\;(-)\right]$	1107.7978	1107.7976	95
$\left[6\;0\;6\;(+)\right]$	−	$\left[5\;2\;3\;(+)\right]$	1107.8319	1107.8315	89
$\left[8\;0\;8\;(-)\right]$	−	$\left[7\;2\;5\;(-)\right]$	1108.6547	1108.6546	86
$\left[8\;0\;8\;(+)\right]$	−	$\left[7\;2\;5\;(+)\right]$	1108.6882	covered	62
$\left[10\;0\;10\;(-)\right]$	−	$\left[9\;2\;7\;(-)\right]$	1109.1992	1109.2000	80
$\left[10\;0\;10\;(+)\right]$	−	$\left[9\;2\;7\;(+)\right]$	1109.2575	covered	61
$\left[12\;0\;12\;(-)\right]$	−	$\left[11\;2\;9\;\;(-)\right]$	1109.3568	1109.3563	85
$\left[12\;0\;12\;(+)\right]$	−	$\left[11\;2\;9\;(+)\right]$	1109.4050	1109.4050	80

## Data Availability

Not applicable.
